# Adapting a Database of Text Messages to a Mobile-Based Weight Loss Program: The Case of the Middle East

**DOI:** 10.1155/2014/658149

**Published:** 2014-01-06

**Authors:** Selma Limam Mansar, Shashank Jariwala, Nawal Behih, Maahd Shahzad, Aysha Anggraini

**Affiliations:** Information Systems Program, Carnegie Mellon University, Doha, Qatar

## Abstract

Obesity has become a worldwide epidemic. Qatar, a rapidly developing country in the Middle East, has seen a sharp increase in the prevalence of obesity. The increase can be attributed to several reasons, including sedentary lifestyles imposed by a harsh climate and the introduction of Western fast food. Mobile technologies have been used and studied as a technology to support individuals' weight loss. The authors have developed a mobile application that implements three strategies drawn from proven theories of behavioral change. The application is localized to the cultural context of its proposed users. The objective of this paper is to present a method through which we adapted the messaging content of a weight loss application to the context of its users while retaining an effective degree of automation. The adaptation addressed body image, eating and physical exercise habits, and regional/cultural needs. The paper discusses how surveying potential users can be used to build a profile of a target population, find common patterns, and then develop a database of text messages. The text messages are automated and sent to the users at specific times of day, as suggested by the survey results.

## 1. Introduction

Tackling the weight issue is a significant undertaking. Worldwide, the number of obese people has doubled in the past 20 years [1]. We explored ways in which mobile technologies can be adapted to meet environmental and cultural norms and thereby support individuals in their effort to lose weight. In this paper, we examine the case of the Middle East through the example of Qatar.

According to the International Association for the Study of Obesity, the numbers for obesity for the Qatari population are alarming. The association ranks the country sixth on its list of the most obese countries worldwide. The numbers presented in [2] (cited by [3]) were overwhelming: for the 25–65 age group, 34.6% of the men were obese and 34.3% were overweight. For females in the same age group, 45.3% were obese and 33% were overweight. The figures are also alarming for children: [4] (cited by [3]) found that in the 12–17 age group, 28.7% of boys were overweight and 7.6% were obese. Additionally, 20.3% of the girls in the same age group were overweight and 4.5% were obese. A more recent study in Qatar [5] suggests slightly lower figures among children of 2–19 years old, but still a much higher percentage than the current 16.9% for American children in this age group as reported by the National Health and Nutrition Examination Survey (NHANES) [6]. Despite this disparity, the media regularly links the nation's weight problem to wealth and also to exposure to Western-style restaurants that allegedly are instilling foreign food habits [7, 8].

The implications of an overweight citizenry for a nation's healthcare system have been widely publicized. A major challenge in addressing the problem has been to find ways to communicate the health implications of overweight and to motivate the population to adopt a healthier lifestyle. The ubiquity of cellphones has attracted the attention of some researchers as both a communication and motivational tool. In Qatar, the mobile phones subscription rate was 142% in February 2012 [9]. Although the Supreme Council of Information and Communication Technology in Qatar (ICTQatar) did not give a specific number, smartphones are said to constitute a significant percentage among the mobile phones in use. The high rate of market penetration of smartphones suggests that users are accustomed to accepting and using mobile applications. Moreover, the results of a study by [10] support the use of mobile applications for weight loss. In their paper, the authors investigated the effects of a mobile phone weight loss program on healthy but overweight adults. The research involved a control group and an experimental group and tested both groups on the use of the mobile phone application. The application instructed participants on how to reduce food intake and take dietary precautions, relayed feedback about goals, and reported daily weight numbers. The outcome of the experiment relied on several variables, all of which indicated at the end of the experiment that the experimental group had lost a significant amount of weight, but the control group had lost only a small amount. The researchers concluded that the mobile phone weight loss program was effective because it helped in propelling and sustaining short-term and long-term weight loss in participants.

Few localized mobile applications on weight loss are available. Brunstein et al. (2012a) [11] conducted a study in the summer of 2012 in which they downloaded the smartphone applications (apps) available through application stores in the “Health and Fitness” category and other health-related categories. Hardly any content in either Arabic or English was found for the residents of the Middle East. The efficacy of customized applications is nevertheless no longer debatable. Many studies over the past decade support localization, at least for websites [12, 13].

An important feature of our application is the messaging between a nutritionist and the users of the mobile application. Using messaging for mobile applications is not new and has been proven to be effective. The messaging (SMS exchange messages) function is usually used to send reminders and motivational or educational messages. Research has proved various degrees of efficacy. For example, shoppers who received advice on food substitution via SMS continued buying healthier alternatives after the program ended [14]. Another study [15] showed that text messages sent to adolescents as part of a diet plan were well accepted, and still another study [16] demonstrated a positive impact from sending weekly e-mails on exercise and food diaries. Authors in [17] reported a similar result from sending biweekly SMSs.

One of the advantages of automated SMSs is the ability to reach many users instantly and deliver information and motivational messages. However, the messages must be well designed to meet the dietary and physical exercise needs of individual users. Indeed, highly personalized support could be achieved by sending differentiated messages to each user, but that would be too time consuming for a nutritionist assisting them. The objective of this paper is to present a method through which we adapted the messaging content of a weight loss application to the context of its users while retaining an effective degree of automation. The adaptation addressed body image, eating and physical exercise habits, and regional/cultural needs.

In this paper, we briefly describe our application, which can be used by any user who can read English or Arabic. Our mobile application uses three features: automated motivational messages and reminders or messaging with a nutritionist, social group support, and self-monitoring of preestablished small and attainable goals. We present the application in Section 2. In Section 3, we discuss our method for creating a well-tailored application for users in a specific region or culture. To prepare the bank of SMS messages, we derived a method that invited input from potential users of the application, then designed the messages, and determined the frequency and timing of their transmission. This method differs from those inother studies in which the acceptance of messages was tested after the experiment [18].  Section 4 presents the results of our method with a group of Arab female students, aged 18–25.  Section 5 concludes with a critical discussion of the results and the method.

## 2. Mobile Application Design

The authors of this paper worked with physicians to understand the nutritional aspect of healthy living in Qatar. In [19], physicians at a large hospital in the country were interviewed. The interviews delivered three consistent messages as follows: (1) most adults and the elderly will prefer communication and technologies presented in Arabic. Young adults and teenagers may be more comfortable with English than their elders, but may still prefer Arabic; (2) most adults and the elderly will need support and guidance in the use of sophisticated technologies; the use of applications has to be straightforward; (3) there is an urgent need for prevention and for raising awareness concerning diets and physical exercise habits.

We also worked with nutritionists and psychologists to understand what may motivate individuals to start a healthful lifestyle or to continue one. Traditional weight loss programs may not trigger long-term change. Multiple simultaneous interventions achieve better results than single-intervention programs; the latter programs typically achieve only modest weight loss. Our analysis led to a description of how theories of behavioral change can be mapped into a mobile application to trigger change [20]. Psychologists advised using Stroebe's theory on behavioral change [21, 22] to address dieters' challenges over short-term intervals. The theory covers three dimensions of dieting.

1. *Cognitive*: it is important to increase dieters' awareness of the goals they set up for themselves. Dieters often have many goals (such as to improve their body image, tone up, lose weight, and cut out fat). Frequent reminders of their goals help maintain dieters' motivation to stay on their program.
2. *Motivational*: the results of neither diets nor physical exercise are immediately noticeable. this delay of gratification may lead to premature abandonment of a program. However, fostering motivation and self-efficacy through the design of more modest goals that can be attained faster helps keep dieters on track. For example, someone can specify a given week or period of time to increase water intake or reduce the consumption of soft drinks.
3. *Social*: dieters live in social environments in which temptations may be too strong. Eating and exercise habits need to be integrated into a social context, possibly one in which friends or relatives support the dieter in achieving his or her goals.

In [20], we proposed a mobile application that supports these three dimensions of behavioral change. To address the *cognitive* aspect, locally designed and tailored SMS messages can be sent daily to remind users of their goals. The *motivational* aspect can be addressed by a design feature in which participants enter incremental, achievable goals weekly. Participants can be asked to indicate daily whether they were successful in reaching each of their goals. In the *social* aspect of the application, social media can be leveraged. Participants can be invited to work toward a collective group goal.

**Figure 1:**
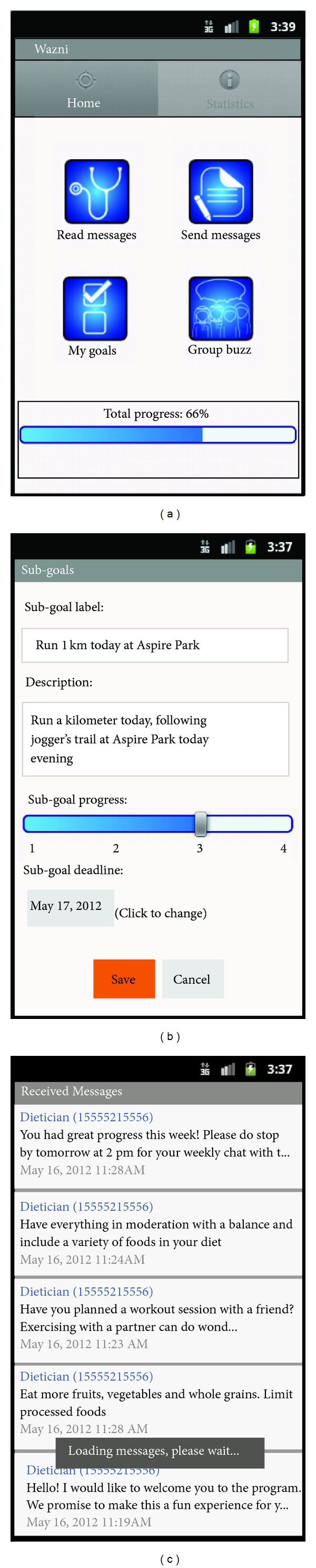
Screenshot of the mobile application (English version).

The application was developed in both English and Arabic, using the Android platform. The usability of the application was tested and fully described in [23].  Figure 1 provides a snapshot of the application's interface and features.

In the following section, we describe the method we used to construct relevant messages.

## 3. A Method for Building Mobile Application Content

The effectiveness of automated messages will be increased if they are tailored to the targeted population group (the individuals pursuing weight loss). By tailoring we mean not only creating meaningful content but also the best times for transmitting this content and the frequency of transmissions as well. Hence, understanding the profile of participants is essential for the optimal customization of the content of these messages. We adopted the following methods.

### 3.1. Survey Design

Our first step was to design a survey to ascertain the most common eating and exercise habits of a targeted population. In the results section, we show the example of targeting a group of female Qatari college students. The survey consisted of questions pertaining to the following

**Figure 2:**
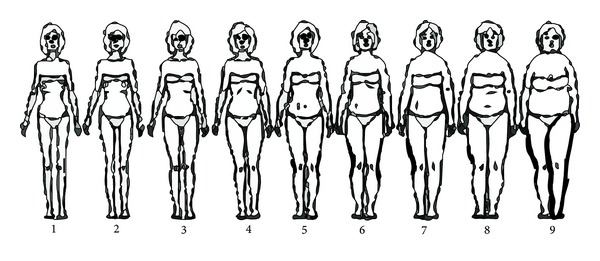
Body contour images used by participants.

1. Demographics.
2. Body mass index (BMI).
3. A contour drawing rating scale (CDRS): it asks respondents to select the outline of a body that they perceive as most closely representing their own as well as one that represents their ideal figure. The CDRS was adapted from [24], Figure 2.
4. Eating patterns and attitudes: this survey section was adapted from a questionnaire by [25]. It serves as a quick guide to identifying dietary habits as well as areas of indulgence or other unhealthful diet or eating patterns (diet type, daily frequency of meals, frequency of eating out, types of restaurants and cuisine, portion size, food pyramid, and beverage intake and type of beverage). An Eating Attitudes Test (EAT) [26] was included to evaluate patterns for behaviors found in anorexia nervosa patients. The test consists of 40 questions reflecting factors such as food habits, perceived body image, vomiting, dieting, and perceived social pressure. Responses were recorded on a 6-point Likert scale: “always,” “very often,” “often,” “sometimes,” “rarely,” or “never.” Reponses were given a score of 3 in the extreme anorexic direction, with adjacent choices given scores of 2 and 1, respectively. All other responses were given a score of zero. A score of more than 30 points indicated symptoms of anorexia nervosa. Participants with the latter score were excluded from the results.
5. Physical activity: this survey section asked respondents if they were physically active and about the amount of time they devoted to physical activity. The section seeks to determine whether respondents fall within the recommended activity levels of the World Health Organization (WHO). WHO defines physical activity in adults (18–64) as including “leisure time physical activity (e.g., walking, dancing, gardening, hiking, and swimming), transportation (e.g., walking or cycling), occupational (i.e., work), household chores, play, games, sports or planned exercise in the context of daily, family, and community activities” [27]. As per [27], we categorized degrees of physical activity as “inactive,” “moderate intensity,” and “vigorous intensity.” The recommendations for healthy adults are a minimum of 30 minutes a day of activity of moderate intensity for five days a week or 20 minutes of vigorously intense activity for three days a week.
6. Typical participant's profile: we used the survey to profile a typical participant's eating and physical exercise behaviors and patterns. The typical profile was used to later design appropriate messages as well as determine the timing and frequency for sending messages.

### 3.2. Database of Text Messages Design

1. We used authoritative references on healthful lifestyles to build a database of messages. We then reduced the messages to short, concise text.
2. We divided the messages into categories. The appropriate categories can be devised after analyzing the survey results and determining the typical eating and physical exercise patterns of a targeted population.
3. We had the selected messages reviewed by a nutritionist to validate the health information.
4. We had the messages reviewed by a psychologist to ensure they were motivational and appealing. Most of the comments from the psychologist related to rephrasing the messages to address small and achievable goals and to convey positive and supportive information.
5. A database of messages was then created. The database arranged the messages according to the frequency and time of day a message from a given category would be sent (daily, weekly, etc.).

### 3.3. System's Architectural Design

See Figure 3.

## 4. Results: Profiling Potential Users in Context

The survey described in Section 3.1 was prepared and distributed by e-mail on September 18, 2012, to students of our university in Qatar. The survey was limited to females currently enrolled as students at the university. Filtering was achieved via the first two questions, which asked whether the responder was enrolled at the university and a female. A response of “No” to either question redirected the responder to the end of the survey. Sixty-eight respondents started the survey; eight of them were male and hence were redirected to the end of the survey. Forty-five of about 174 female students (52% of a total of 335 degree-seeking students) completed the survey, which was closed on September 29, 2012. This yields a response rate of 26%; all respondents are considered female students at the university. However, the total number of female native Arabic speakers at the university is not available.

### 4.1. Identifying Demographics, BMI Figures, and Body Image

Survey responses were further filtered, by selecting those who were native speakers of Arabic. These 26 responses were analyzed. About 65% of the native Arabic-speaking female students were 18–20 years old. The mean BMI of this group was 24.65 (which is in the normal range, overweight starting at 25 or above), in the range of 17 (underweight)–41.5 (obese class III); median and mode were 25.4 (overweight) and 27.1 (overweight), respectively, with a standard deviation of 5.62 (see Table 1). A strong correlation (0.87) was observed between the respondents' BMI and the CDRS figure they considered most like themselves. This suggests that most respondents were aware of their current weight and body figure: a BMI in a normal range translated to a normal range on the CDRS. However, one out of three obese participants and four of 11 who were overweight underestimated their body image. A similar lack of self-awareness was evident in one of the studies assessing the relationship between BMI and body image self-perception [28]. Another finding that corroborates with self-perception studies is that even females with BMI in the normal range (18.5–24.9 kg/m^2^) would ideally like to lose weight [29]. Of the nine respondents with BMIs in the normal range, seven wanted to lose weight to ideally move further down the CDRS. Furthermore, none of the respondents marked the middle image on the CDRS as their ideal goal image. The highest selection was 4 on the 9-point scale, a selection still regarded as anorexic [24].

### 4.2. Identifying Eating Patterns and Attitudes

The most common diet was “halal” food, comparable to a kosher diet in the West. This is a religious and cultural norm, and almost all the respondents followed this particular diet. Of the 26 respondents, only two followed a low-fat diet, and two followed a vegetarian diet. These respondents with special diets, such as low fat, restricted their eating to at least two hours before sleeping or received specially planned meals from a diet shop. These persons were slightly above the normal BMI threshold and perceived their body images as normal (4-5, on a 9-point scale).

In terms of meals consumed, respondents on average consumed about two meals a day, with lunch—out of breakfast, brunch, lunch, and dinner—being the most frequent meal consumed. A large proportion of the group snacked in the afternoon (12) or throughout the day (12). The most common snacks included chocolate, chips, crackers and biscuits, cookies, cereals, and fruits.

An indicator of cultural eating habits may be the frequency with which young adults eat out or order “takeout” meals in [30]. We found that 23 of the 26 respondents ate out or had food delivered; 13 of them did so at least once a week. The most common dining facilities frequented were fast food restaurants (14), followed by eating at home and in the university cafeteria. The fast food option is not good news. As indicated in [31], regular fast food consumption leads to a steady increase in calories intake and hence to weight gain. The most popular cuisines were Italian, American, and Lebanese, followed by Chinese, Mexican, Indian, and traditional Qatari cuisines. However, there was no clear correlation between BMI and eating facilities or cuisine. The two most common food preparation techniques were frying (20) and boiling (20), followed by baking (19) and steaming (14).

Respondents were asked how frequently they ate various types of food, such as starch, dairy, meat, poultry, and fat.  Table 2 shows the responses. The results are inconclusive. Indeed, the sizes of food portions were not captured and could play a larger role than just that of the frequency of intake [32]. Furthermore, the frequency of starch intake does not disclose the type of starch consumed, which could be either beneficial slow-digesting starch or undesirable starch sugars high in fructose, such as high fructose corn syrup, or even both [33].

Respondents were asked about their intake of beverages. We compared the consumption of water with the suggested adequate intake (AI) of water. Adequate intake is the average total water intake, including direct and indirect water consumption, by a group of healthy people [34]. The AI for young adult females is 2.7 L [35], a little over 11 glasses (8 fl. oz. each). Another category in which frequency may not translate to consumption is tea and coffee intake. Eighteen of 26 respondents have either tea or coffee 1–5 times a day, with five having both. The question does not capture how much sugar is added to these beverages, and high sugar content in beverages is detrimental even at 1–5 cups a day. Tea in this region, especially the local “karak” tea, which contains high amounts of sweetened condensed milk, typically contains a large amount of sugar.

The respondents were asked if they would like to change their eating habits, and 23 of 26 responded “Yes.” The most common changes the respondents wanted to bring about were replacing unhealthful snacks with more healthful options, cutting down on junk food, reducing sugar intake, and making breakfast a regular daily meal, along with balanced meals in general (see Table 3).

The final segment of the survey included the Eating Attitudes Test (EAT) (see Section 3.1). From a total of 26 eligible respondents, four scored more than 30 on the EAT; of these, only one was underweight (BMI 17.2, EAT 41) and one of the participants had a BMI within the normal range (BMI 23.8, EAT 36). It is noteworthy that two participants were overweight, yet nevertheless scored high on the EAT (BMI 27.1, EAT 61 and BMI 28, EAT 41). We then consulted their responses on the 9-point CDRS to what they would ideally like to resemble [24]. Each of the participants had entered a selection corresponding to the lower range of the scale (2, 3, and 4) in which a selection of up to 4 correlated with symptoms of anorexia. Hence, we see that even healthy, or slightly overweight, females are expressing signs of anorexia. Healthy females scoring over 30 might be undergoing significant concerns regarding their body image and weight.

We believe that these behaviors—attitudes toward eating and concerns about body image in healthy or overweight females—may be attributable to the local social stigma pertaining to weight and body image. In the region, having a thin figure is part of what is deemed attractive. This puts social and peer pressure on females to “fit” the image of an attractive young woman. Young females are constantly concerned about their body image, and we find that even healthy females are dissatisfied with their perceived body images and want to lose weight (see the section on BMI-image dissatisfaction). These social and peer pressures, along with the social definition of attractive, may be the factors behind body image dissatisfaction and anorexic health behaviors and attitudes toward food (see Table 4).

### 4.3. Identifying Physical Exercise Patterns and Habits

The third section of the survey asked respondents if they were physically active (see Section 3.1). Ten of the 26 respondents were inactive; among those who were active, a majority (10) engaged in only 21–40 minutes of vigorous intensity per week (Table 5). Four respondents engaged in less than 30 minutes, 31–60 minutes, and 61–90 minutes of physical activity of moderate intensity (Table 6). Twenty-two respondents felt that they must change their physical activity habits and would like to do so by exercising more, engaging in some sorts of sports, enrolling in a gymnasium, or introducing fast walking into their daily routine.

Most of the respondents fell far short of the recommended amount of physical activity (see Table 5).

### 4.4. Deriving a Typical Respondent (Native Arabic Speaker) Profile

We targeted female respondents enrolled in our university who are native speakers of Arabic. We had a total of 26 respondents from the target group after filtering out male respondents, those who were not currently enrolled, or those who were not native Arabic speakers.

The typical respondent, based on the survey, is a female native speaker of Arabic who is a student in our university and between the ages of 18 and 20. She has a normal range BMI of 24.65 (her self-perceived body image is 4.23, and her desirable body image is 2.92 on a 9-point scale).

In terms of eating patterns, she follows a halal diet. Her most regular meal of the day is lunch, with breakfast being the meal most often skipped. She also snacks throughout the day; her typical snacks are chocolate, chips, crackers, or biscuits. She eats out or orders takeout meals at least once a week, and fast food is typically her first choice. American or Italian is her usual cuisine of choice. She balances various types of food preparation, such as frying, boiling, and baking, but does not typically broil. In terms of food items, her diet is balanced between starch, vegetables, dairy, meat, fat, and sweets. She should, however, increase her daily fruit intake. In terms of fluid consumption, she does not consume alcohol, but she would benefit from reducing her intake of soda and juice and also by drinking 3 to 7 more glasses of water a day. She would like to change her eating habits, starting by replacing unhealthful snacks with healthier options, cutting down on fast food, reducing her sugar intake, and having balanced meals, a goal that includes making breakfast a regular part of her day.

This typical respondent is also moderately active physically. She does not engage in enough physical activity and would benefit from significantly increasing it. She would also like to change her exercise habits by engaging in activities with more vigorous intensity, increasing the weekly frequency of her workouts, enrolling in a gymnasium, and introducing fast walking into her physical activity. Finally, in terms of her eating attitudes, she does not exhibit symptoms of anorexia nervosa.

### 4.5. Building a Database of Text Messages

We used the survey results to compile a database of messages. The categories for the SMS database were discerned from the survey, based on the topics that needed to be addressed. The category of “eating habits” contained messages targeting a change in eating habits, such as having balanced meals, eating at a comfortable pace and within limits, fighting urges, and replacing sugary beverages with water. A majority of the survey respondents wanted to overcome unhealthful habits, to cut down on sugar, and to eat healthful and balanced meals. A category of junk food was introduced as well. It aimed at educating the participants on the detrimental effects of junk food and at suggesting healthier alternatives, reducing the frequency of eating junk food, and suggesting alternative food preparation techniques, such as grilling or broiling instead of frying. As we have seen in the eating patterns discerned from the survey, more than half of the respondents go to fast food restaurants and would like to switch to healthier options. Consequently, we included a category on restaurants to induce healthful eating behavior when eating out on weekends with friends or family. Typical messages suggested replacing side dishes with healthier options, such as replacing fried or mashed potatoes with a salad, replacing soda with water, sharing a dessert, and starting meals with soup and salad. Thirteen of the 23 respondents who ate out or ordered takeout meals did so at least once a week, and this is another instance of behavior that we can change for the better. We introduced a category on snacks to help participants make better choices instead of trying to stop snacking entirely. The messages promote a healthier approach to snacking, such as fighting the urge to snack all the time, avoiding snacks before bedtime, keeping healthier alternatives such as fruits in sight, and doing diet-conscious grocery shopping, such as picking up water-filled grapes because they occupy more stomach volume. Finally, a category on physical activity was included because, as mentioned earlier, almost all the respondents were far short of the recommended amount of physical activity. Moreover, most of the respondents, as is the case with a majority of the population in the region, lead sedentary lifestyles with insufficient outdoor physical activity because of the unfavorable climate. Most of the respondents, as is the case with a significant number of students here, are dropped off or picked up close to the doors of the university. Hence, our messages include such suggestions as parking farther away than usual at the university and also at shopping malls and taking stairs instead of elevators. Other messages encourage working out, visiting a gymnasium regularly (with friends, to keep up motivation), as well as general messages calculated to raise awareness of the benefits of exercise (see Table 7).

## 5. Conclusions: Lessons Learned and Future Guidelines

This paper presented a method for designing text messages for use in a contextual mobile application. The application is designed to support achieving sustainable weight loss. With our method, text messages are not guesses aimed at targeting potential future users but are derived after creation of a profile of typical users. This profile was constructed through the use of a survey that adapted previous research into an overall design to elicit respondents eating and exercise habits as well as gain insights into their relationships to food and their perceptions of their bodies. After the typical user profile was constructed and the database of messages was developed, the messages were reviewed by a nutritionist and a by psychologist to validate their health information and to ensure they were motivational.

To illustrate our method, we ran the survey with the targeted background of young native Arabic-speaking females at our university. We analyzed their eating patterns and typical health behavior. Some of the important findings showed that the typical representative of the target population does not eat healthfully, skips meals while still maintaining a balanced diet, does not consume enough water, and does not engage in recommended amounts of physical activity. These findings allowed us to develop a customized database of text messages that can be used in a mobile application. The mobile application can be used to transmit timely text messages aimed at the nutrition and exercise habits of a typical respondent profile.

The results presented are localized to young Arab females attending universities. The derived profiles can be of interest to universities in the region as well as nutritionists concerned about healthful habits in the Middle East. However, our method does not depend on the context of the study and may be used to assess respondents' eating and physical activity profiles. Even if used on a nonhomogeneous group (say patients attending a weight loss clinic of mixed genders, ages, and ethnicities), it is possible, through statistical analysis of the surveys, to derive profiles and adapt sets of text messages. The survey would identify not just one but as many profiles as the studied group would suggest it encompasses.

The next step in our research will be to test the application as well as its culturally adapted content through a five-week pilot study. The study will test the effectiveness of each of the three components—the SMS exchange, goal setting and progress monitoring, and social support network—of the mobile application against traditional intervention methods. The usability questionnaires, distributed exclusively to the experiment group that uses smartphones, will help assess the adaptability of the mobile application to the local context and culture. On the other hand, the health behavior questionnaires, given to both the experiment and the control groups, will help track participants' changes in health behavior and attitudes; they also will yield qualitative information, such as temptations encountered and how participants overcame them.

A future study lasting 15 weeks would permit firmer conclusions about the application's effectiveness because it will enable measurement of behavioral changes and weight loss over a longer duration. This will help assess any sustainable weight loss and behavioral change. It will also enable researchers to evaluate each aspect of the mobile application and hence the underlying theories of behavioral change.

## Figures and Tables

**Figure 3:**
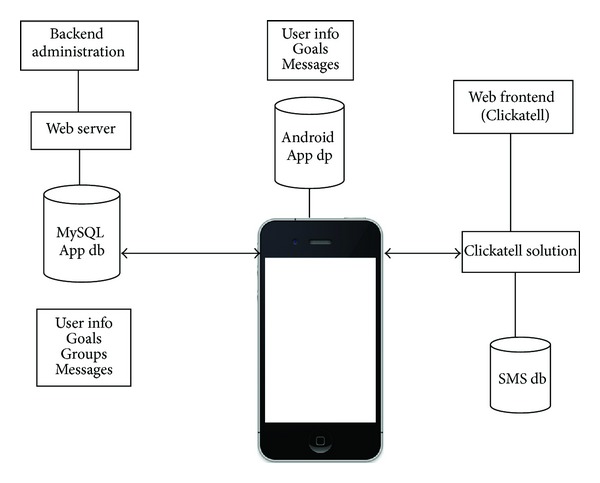
Application architecture.

**Table 1:** BMI profiles.

BMI range	Average	Median	Mode	Standard deviation	Perceived CDRS mode	Desirable CDRS mode
17–41.5	24.65	25.4	27.1	5.62	4	3

**Table 2:** Eating patterns: food items.

Item	Freq.					
	Never	1-2	3–5	6–8	9–11	Total
Starch (bread, rice, pasta, and potato)	0	15	8	3	0	26
Fruits	0	20	6	0	0	26
Vegetables	0	16	10	0	0	26
Dairy	2	19	3	2	0	26
Meat, fish, poultry, and eggs	0	15	8	2	1	26
Fat	2	16	5	3	0	26
Sweets	1	18	5	2	0	26

**Table 3:** Beverages.

Item	Freq.			
	1–5	6–10	Not consuming	Total
Water (glasses)	20	6	0	26
Coffee	10	0	16	16
Tea	13	0	13	26
Soda	7	0	19	26
Alcohol	0	0	26	26
Others (juice, etc.)	6	0	26	26

**Table 4:** EATs values.

Range	Average	Median	Mode	Standard deviation	Cut-off score	Score greater than 30
5–61	21.85	21.5	14	12.93	30	4

**Table 5:** Vigorous-intensity physical activity.

Estimated engagement in vigorous-intensity physical activity (minutes per week)					
<20	21–40	41–60	61–75	>75	Physically inactive
3	7	4	1	1	10

**Table 6:** Moderate-intensity physical activity.

Estimated engagement in moderate-intensity physical activity (minutes per week)						
<30	31–60	61–90	91–120	121–150	>150	Physically inactive
4	4	4	3	0	1	10

**Table 7:** SMS categories and frequency.

Category	Number of messages	Frequency	Typical timings	Sample message
Eating habits: driving change	23	Daily	Breakfast (7:15 am) Lunch (11:30 am) Dinner (8:00 pm)	A calorie is a calorie regardless of its source. Whether you're eating carbohydrates, fats, sugars, or proteins, all of them contain calories [36]
Junk food: alternatives and awareness	22	At least four times a week	Lunch (12:00 pm) on Friday Evening (6:00 pm) on Saturday Alternating lunch and evening on weekdays	Fast foods have no nutritional value. “They are very low in vegetables. Most of it is refined products and processed foods” [37]
Restaurants: alternatives and awareness	6	Weekends (Thursday, Friday)	Evening (6:00 pm)	Start your meal with a soup and salad and order vegetables as your side dish [38]
Snacks: good choices	30	Daily	Morning (10:30 am) Afternoon (3:00 pm) Evening (5:30 pm) Night (9:00 pm)	Having snacks in a convenient place to reach is helpful. Try putting fruit in a bowl on the counter, so you can grab an apple or orange when you're hungry [39]
Physical activity:	26	Daily	Morning (9:00 am) Afternoon (4:00 pm)	Hunt for the farthest parking space. If you drive to run errands, purposefully park your car a little farther from your store entrance [40]
